# Sternal Complications After Clamshell Surgery for (Heart-)Lung Transplantation—A Systematic Literature Review

**DOI:** 10.1093/ejcts/ezaf318

**Published:** 2025-09-23

**Authors:** Dorine S Klei, Rengin Sabaoğlu, Mostafa M Mokhles, Linda M de Heer, Karlijn J P van Wessem

**Affiliations:** Department of Trauma Surgery, University Medical Centre Utrecht, 3584 CX Utrecht, The Netherlands; Department of Cardiothoracic Surgery, University Medical Centre Utrecht, 3584 CX Utrecht, The Netherlands; Department of Cardiothoracic Surgery, University Medical Centre Utrecht, 3584 CX Utrecht, The Netherlands; Department of Cardiothoracic Surgery, University Medical Centre Utrecht, 3584 CX Utrecht, The Netherlands; Department of Trauma Surgery, University Medical Centre Utrecht, 3584 CX Utrecht, The Netherlands

**Keywords:** clamshell thoracosternotomy, bilateral lung transplantation, heart-lung transplantation, sternum, complications, systematic review

## Abstract

**Objectives:**

Bilateral transverse thoracosternotomy (“clamshell”) is widely used for (heart-)lung transplantations, but postoperative sternal complications are a significant challenge. The primary objective of this systematic review was to evaluate the prevalence of sternal complications after clamshell surgery in (heart-)lung transplantation patients.

**Methods:**

A systematic literature review was conducted. On April 4, 2025, PubMed and Embase databases were searched. Original studies reporting sternal complications after clamshell surgery in adults for bilateral lung or heart-lung transplantation were included. Studies including <10 patients were excluded. Study quality was assessed using the National Institutes of Health assessment tool. The total and range of sternal complication prevalence was provided. Meta-analysis of sternal complication prevalence was not performed due to significant heterogeneity across studies.

**Results:**

The database searches yielded 945 eligible articles. Eighteen studies were included, including 828 patients who underwent a total of 830 bilateral lung or heart-lung transplantations through clamshell surgery. All included studies were cohort studies with poor (*n* = 15), fair (*n* = 1), or good (*n* = 2) quality. In total, 286 sternal complications were reported (0.34 event per clamshell surgery; range 0.02 to 1.35 in individual studies) and 90 sternal reoperations were conducted (0.14 reoperation per clamshell surgery; range 0.02 to 0.29 in individual studies).

**Conclusions:**

Despite limitations in study quality and heterogeneity, this review highlights the high prevalence and relevance of sternal complications following clamshell surgery for (heart-)lung transplantation. Future studies should focus on patient selection, risk stratification, development of modified sternal closure techniques, and implementation of alternative surgical approaches to (heart-)lung transplantation.

## Graphical Abstract

**Figure:**
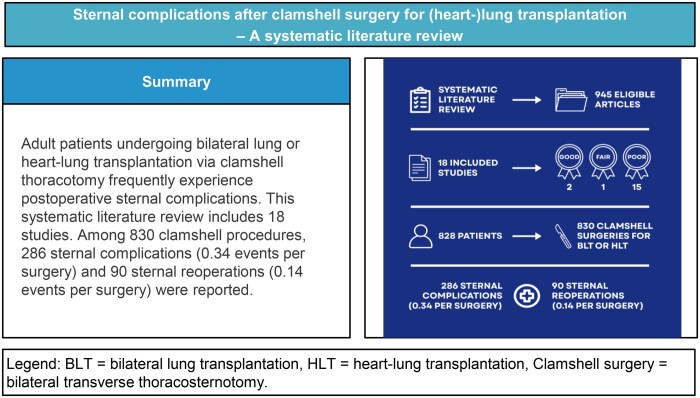

## INTRODUCTION

The clamshell approach, a bilateral transverse thoracosternotomy usually performed in the third, fourth, or fifth intercostal space, is the conventional surgical approach for bilateral lung transplantation and sometimes also used for heart-lung transplantation.^1–9^ Although the clamshell approach provides optimal exposure of thoracic organs,^2–4^^,^^8–14^ it is associated with significant sternal complications including sternal pseudoarthrosis (non-union), sternal dehiscence, and infection.^1^^,^^3–5^^,^^7^^,^^8^^,^^15^^,^^16^ These complications can lead to sternal instability, chest deformity, chronic pain, and impaired chest wall function, thereby causing substantial morbidity often requiring reintervention.^1^^,^^3–5^^,^^7^^,^^15^ Risks of these complications are increased due to extensive surgical trauma caused by the clamshell approach^1^^,^^4^^,^^12^^,^^15^^,^^17^^,^^18^ in combination with comorbidities in (heart-)lung transplantation patients, such as diabetes, malnutrition, and chronic use of immunosuppressive medication.^1^^,^^5–8^^,^^15^^,^^19–21^

In literature, the prevalence of postoperative sternal complications ranges widely between near-zero to 60%, and depends on definition of complications and primary sternal closure techniques.^1^^,^^3^^,^^7^^,^^18^^,^^19^ The sternum is traditionally closed with wire cerclage (2-3 wires),^3^^,^^12^^,^^19^ although alternative techniques have been proposed to decrease sternal complications, such as crossed wiring,^3^ sternal plating,^1^ and FiberTape.^5^

While many studies report short-term complications after the clamshell approach, very few report long-term sternal outcomes.^3^^,^^7^^,^^22^ To our knowledge, no systematic review of literature has ever been conducted on (short-term or long-term) sternal outcomes after clamshell thoracotomy. Therefore, this systematic literature review aimed to quantify the prevalence of sternal complications after clamshell incision for bilateral lung or heart-lung transplantation, to serve as benchmark for future studies on sternal complications and alternative surgical approaches.

## METHODS

### Study design

A systematic literature review was conducted according to the PRISMA guidelines.^23^ Ethical approval was not required as this study was based on published literature.

### Search strategy

Literature search was performed using the PubMed and Embase databases, searching for articles reporting sternal outcomes after clamshell surgery in adult bilateral lung transplantation or heart-lung transplantation patients, from database inception to April 4, 2025. The search strings contained free, controlled, and Mesh/Emtree terms (see **Supplementary Material**). Snowballing was conducted by screening reference lists of all included studies and relevant review articles identified during the screening process.

Randomised controlled trials, cross-sectional studies, case-control studies, cohort studies, and case series (including 10 or more patients) were included if they reported sternal complications after clamshell surgery in adult bilateral lung or heart-lung transplantation patients. Articles were excluded for different surgical approach, different patient population than bilateral lung and/or heart-lung transplantation patients, animal or cadaver studies, laboratory studies, case series reporting less than 10 patients, case reports, conference abstracts, paediatric patients, or language other than English or Dutch. If patients undergoing clamshell surgery for (heart-)lung transplantation comprised subgroups of a study, this study was included if sternal outcomes were reported separately for such subgroup.

### Selection process

Two authors (R.S. and D.S.K.) independently performed study selection, screening titles and abstracts, and thereafter assessing full-texts of eligible studies. A consensus meeting with a third reviewer was not necessary since no disagreement occurred.

### Data collection process and data items

R.S. and D.S.K. independently extracted and cross-checked data points from each study: study characteristics (authors, publication year, centre, country, study design, inclusion period, number of patients, number of clamshell surgeries, follow-up duration); patient characteristics (age during surgery [years], sex, aetiology, body mass index [BMI]); patient comorbidities (pre-existent osteoporosis, diabetes, smoking, malnutrition, use of preoperative immunosuppressive medication, previous same-site surgery); perioperative outcomes (operative time [minutes], intraoperative cardiopulmonary bypass [CPB]/extracorporeal membrane oxygenation [ECMO] use, duration of postoperative drainage [days], length of hospital and intensive care unit [ICU] stay [days]); sternal closure technique; and sternal complications. Evaluated sternal complications included pseudoarthrosis (non-union), sternal dehiscence (including sternal separation and override), sternal wire problems (wire migration and other mechanical problems), sternal infection (superficial or deep wound infection; infected haematoma; abscess; osteomyelitis), sternal tissue necrosis, sternal instability, sternal malunion, sternal deformity, chronic sternal pain, sternal clicking sensation, and restricted physical activity due to sternal complications. Sternal reoperation was subdivided into reoperation for sternal complications or long-term removal of sternal osteosynthesis material. The definitions and criteria used by the studies were followed, providing a complete overview of reported sternal complications. For chronic postoperative pain, if location was not specified, it was assumed to be partly/fully located at the sternotomy.

Additionally, articles were assessed for any data regarding the correlation between preoperative patient characteristics and sternal complications, and the correlation between sternal complications and other (long-term) treatment outcomes.

In case of incomplete reporting, patient numbers were assumed equal to the number of surgeries. Ambiguities in reported outcomes, such as when the number of complications or reoperations could not be fully determined, were addressed by denoting minimum values (indicated as “Min.” in tables and “At least” in text). This approach aimed to reduce potential underestimation due to selective reporting.

Continuous variables were collected using statistical units provided by the reporting article (mean, median, standard deviation, interquartile range [IQR] and/or range).

### Risk of bias assessment

Study quality was assessed using the National Heart, Lung and Blood Institute of National Institutes of Health (NIH) quality assessment tool for observational cohort studies.^24^ Two criteria (levels of exposure and multiple exposure assessments) were removed because they were not applicable. Each remaining criterion was scored as “Yes,” “Unclear,” or “Not applicable” by R.S. and D.S.K. independently. In case of disagreement, the authors reviewed the study together and consensus was reached in all cases without a third reviewer’s verdict.

Each criterion was awarded 1 point when answered with “Yes,” leading to a maximum of 12 points per study. “Good” quality ratings were awarded to studies with 10 points or more, “Fair” quality for 7 to 9 points, and “Poor” quality for under 7 points.

### Data analysis

The primary end-point was prevalence of sternal complications, reported and tabulated as total number of complications (potentially exceeding the number of patients). All studies reporting a specific complication were included in data synthesis for that complication.

Based on the total number of complications, number of complications per clamshell surgery and number of surgeries per complication were calculated.

Due to significant heterogeneity across studies regarding types and definitions of reported sternal complications, no subgroup-analyses, meta-analyses, heterogeneity assessments, or sensitivity-analyses were performed, and certainty of evidence was not formally assessed. Instead, a descriptive synthesis of the range of reported prevalences was provided.

Data analysis was performed using IBM SPSS-Statistics version 29.0.1.0(171).

## RESULTS

### Literature search

After removing duplicates, 1,006 articles were identified through the database searches in PubMed and Embase. After screening titles and abstracts of these articles, 143 articles were included for full-text screening. No full-text was available for 6 articles.

**Figure 1.**
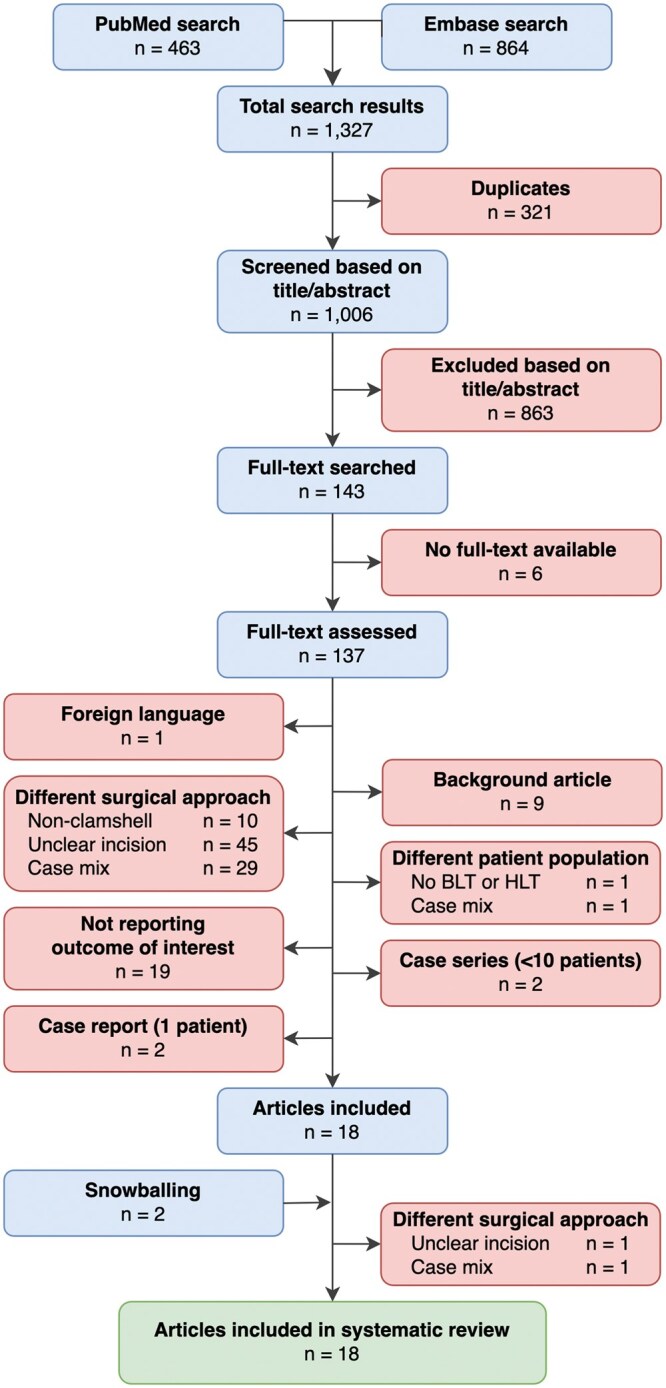
Study Selection Flowchart. Abbreviations: BLT, bilateral lung transplantation; HLT, heart-lung transplantation. “Case mix,” no separate outcomes reported for the subgroup undergoing clamshell surgery (surgical approach) or the subgroup undergoing BLT and/or HLT (patient population)

Of the remaining 137 articles, 119 were excluded because of foreign language (*n* = 1), background article (*n* = 9), different surgical approach (*n* = 84), different patient population (*n* = 2), not reporting outcome of interest (*n* = 19), case series <10 patients (*n* = 2), and case reports (*n* = 2). Snowballing yielded 2 additional articles for full-text assessment; both articles were excluded for different surgical approach. The remaining 18 studies met the inclusion criteria for this review (**Figure 1**).

### Study characteristics

The included studies yielded 828 patients, who underwent 830 transplantations through the clamshell approach (some patients underwent retransplantation). Of these, 817 patients underwent bilateral lung transplantation and 10 a heart-lung transplantation. Among the 18 included articles was 1 prospective cohort study, 16 retrospective cohort studies, and 1 unclear cohort study. All studies were monocentre studies and comprised distinct study populations. Clamshell surgeries were conducted between 1989 and 2022. Follow-up duration was mostly unclear, and ranged from 1 to 103 months. A detailed overview of study characteristics is presented in **Table 1**.

**Table 1.** Characteristics of Included Studies^a^

Study	Country	Study design	Inclusion period	No. of patients included	No. of clamshell surgeries conducted	No. of patients undergoingBLT	No. of patients undergoing HLT	Primary sternal closure technique	Follow-up duration for sternal complications	Study quality
Boudreaux et al. (2021)	United States	Retrospective cohort	January 2016 - June 2020	22	22	22	0	Wiring (2 wires) or plate-and-band rigid fixation	Unclear	Poor
Brown et al. (1996)	Australia	Cohort of unclear type	Unclear	31^b^	31	31	0	Wiring (2-3 wires) or 2-plate system	Unclear	Poor
Coloni et al. (2004)	Italy	Retrospective cohort	Unclear; approx. 1996 - 1999	32^b^	32	32	0	NR	Unclear	Poor
Costa et al. (2015)	United States	Retrospective cohort	2006 - 2013	106^b^	106	106	0	Reinforced wiring	Min. 60 days	Poor
Coster et al. (2022)	United States	Retrospective cohort	January 2021 - January 2022	50	50	50	0	Reinforced wiring or FiberTape	Unclear	Poor
Elde et al. (2017)	United States	Retrospective cohort	July 2013 - June 2016	68^b^	68	68	0	NR	Unclear	Fair
Force et al. (2006)	United States	Retrospective cohort	January 2003 - March 2005	15	15	15	0	Wiring (3 wires)	Unclear	Poor
Fuller et al. (2018)	Australia	Prospective cohort	Unclear	20	20	20	0	NR	Min. 1 month	Poor
Kaiser et al. (1991)	United States	Retrospective cohort	October 1989 - January 1991	27	28	28	0	Wiring	Range 2-15 months	Poor
Klinger et al. (2020)	United States	Retrospective cohort	July 2014 - September 2016	54	54^b^	54^b^	0	NR	Min. 2 months	Poor
Koster et al. (2013)	The Netherlands	Retrospective cohort	January 2001 - February 2011	129	129	126^c^	0	Uncrossed wiring or (para)sternally crossed wiring	12 months	Good
Macchiarini et al. (1999)	Germany	Retrospective cohort	January 1990 - March 1998	37	37	27	10	NR	Mean 3.7 years (± 2 years)	Poor
Meyers et al. (1999)	United States	Retrospective cohort	April 1995 - December 1997	50	51	51	0	NR	Mean and median 2.5 years	Poor
Motomura et al. (2011)	United States	Retrospective cohort	January 2007 - May 2009	59	59^b^	59^b^	0	Wire cerclage	Min. 6 months	Poor
Olland et al. (2017)	France	Retrospective cohort	January 2016 - October 2016	14	14	14	0	Sutures or absorbable sternal pins	Unclear	Poor
Oto et al. (2007)	Australia	Retrospective cohort	March 2004 - June 2006	70	70	70	0	Conventional wiring or reinforced wiring	6 months	Good
Park et al. (2023)	South Korea	Retrospective cohort	May 2013 - December 2017	34	34	34	0	Wiring (2 wires) or plate fixation	Unclear	Poor
Wong et al. (2008)	China	Retrospective cohort	July 1995 - December 2006	10	10	10	0	NR	Mean 55 months (range 1-103)	Poor
**Total (all included studies)**				**828**	**830**	**817**	**10**			

Abbreviations: BLT, bilateral lung transplantation; HLT, heart-lung transplantation; Min., minimally; No., number; NR, not reported.

a Study characteristics not (clearly) reported, are marked as “Unclear.”

b If only patient number or number of surgeries was reported, the other was assumed to be equal.

c In 3 patients, bilateral lung transplantation was converted to single lung transplantation.

### Risk of bias assessment

Fifteen studies had poor quality, 1 study had fair quality, and 2 studies had good quality (**Table 1**; **Table S1**). Most notably, few studies provided a clear definition of outcome measures and the criteria and time frame for assessment of these outcomes, which raised uncertainty regarding the consistent implementation and examination of outcome measures across all study participants.

### Preoperative characteristics

The percentage of female patients ranged from 32.0% to 75.0%.^1^^,^^3–5^^,^^15^^,^^17^^,^^21^^,^^25–27^ Mean age varied from 40 to 54 years.^1^^,^^3^^,^^4^^,^^15^^,^^17^^,^^21^^,^^25–27^ Chronic obstructive pulmonary disease (COPD),^1^^,^^3^^,^^5^^,^^15^^,^^17^^,^^21^^,^^25^ cystic fibrosis,^1^^,^^3^^,^^17^^,^^21^^,^^25^^,^^26^^,^^28^ and interstitial lung disease^1^^,^^3^^,^^15^^,^^26^ were the aetiologies reported by most studies. Additional preoperative variables reported by included studies are presented in **Table S2**.

### Perioperative characteristics

Mean operative time ranged from 320 to 745.18 minutes.^1^^,^^3^^,^^15^ Use of intraoperative CPB/ECMO varied between 29.6% and 100.0% of patients.^1^^,^^3^^,^^4^^,^^7^^,^^15^^,^^17^^,^^26^^,^^27^ Duration of postoperative drain use was never reported. Mean length of hospital stay ranged from 24 to 74.32 days,^15^^,^^17^^,^^26^ while mean ICU length-of-stay varied from 4.1 to 21.54 days^3^^,^^15^^,^^17^^,^^26^ (**Table S2**).

### Sternal closure technique

Eleven studies described their sternal approximation technique, varying from sternal wire cerclage^1^^,^^3^^,^^5^^,^^15^^,^^19^^,^^21^^,^^22^^,^^26^^,^^29^^,^^30^ (by some authors further subdivided into uncrossed wiring,^3^ crossed wiring,^3^^,^^30^ or reinforced wiring^5^^,^^19^^,^^21^) to absorbable sternal pins,^6^ sutures,^6^ plate fixation,^15^^,^^22^ plate-and-band rigid fixation,^1^ and FiberTape.^5^

Six studies^1^^,^^3^^,^^5^^,^^6^^,^^21^^,^^22^ compared and reported results of different primary sternal re-approximation techniques. One study^15^ compared different closure techniques but did not report separate results. Details of closure techniques varied considerably between authors; therefore, pooling of subgroups was impossible.

Generally, alternative sternal closure techniques tended to result in less complications than sternal wiring-cerclage. Boudreaux et al reported complication rates of at least 2/19 with plate-and-band fixation (0.11 event per surgery), but 3/3 with wire-cerclage (1.00 event per surgery).^1^ Similarly, Brown et al reported 5 complications in 11 surgeries with wire-closure (0.45 event per surgery), but only 1 in 20 with his 2-plate closure-system (0.05 event per surgery).^22^ Coster et al reported similar results, comparing reinforced wiring (15/28, 0.54 event per surgery) with FiberTape closure (0 complications in 22 surgeries).^5^

Another study assessed different wiring techniques and reported complications rates of 44/79 (0.56 event per surgery) for uncrossed wires, versus only 1/17 (0.06 event per surgery) with a parasternally crossed wiring-technique.^3^ Oto et al reported similar complication rates comparing conventional wiring (22/43, 0.51 event per surgery) and reinforced wiring (13/27, 0.48 event per surgery).^21^

Zero complications were reported with absorbable pins closure compared with 2 events per surgery in the control group, but this control group consisted of 2 patients and closure technique was unspecified.^6^

### Sternal complications

#### Overall sternal complications

At least 286 sternal complications occurred across 830 clamshell surgeries, corresponding to 0.34 complications per surgery or 2.9 surgeries performed for one sternal complication (**Table 2**). The number of complications per conducted surgery ranged from 0.02^19^ to 1.35^25^ in individual studies (**Table 2**).

**Table 2.** Sternal Complications per Study

Study	**Number of clamshell surgeries conducted** ^a^	**Total number of sternal complications** ^b^	Number of sternal complications per conducted surgery	Number of conducted surgeries per sternal complication	**Number of sternal reoperations due to complication** ^c^	Number of sternal reoperations per conducted surgery
Boudreaux et al. (2021)	22	6	0.27	3.7	Min. 3	0.14
Brown et al. (1996)	31	6	0.19	5.2	–	–
Coloni et al. (2004)	32	3	0.09	10.7	–	–
Costa et al. (2015)	106	Min. 2	0.02	53.0	2	0.02
Coster et al. (2022)	50	15	0.30	3.3	Min. 8	0.16
Elde et al. (2017)	68	Min. 12	0.18	5.7	15	0.22
Force et al. (2006)	15	1	0.07	15.0	1	0.07
Fuller et al. (2018)	20	Min. 27	1.35	0.7	–	–
Kaiser et al. (1991)	28	5	0.18	5.6	–	–
Klinger et al. (2020)	54	31	0.57	1.7	–	–
Koster et al. (2013)	129	56	0.43	2.3	Min. 10	0.08
Macchiarini et al. (1999)	37	22	0.59	1.7	Min. 8	0.22
Meyers et al. (1999)	51	Min. 26^d^	0.51	2.0	11	0.22
Motomura et al. (2011)	59	Min. 25	0.42	2.4	4	0.07
Olland et al. (2017)	14	Min. 4	0.29	3.5	2	0.14
Oto et al. (2007)	70	35	0.50	2.0	20	0.29
Park et al. (2023)	34	Min. 9	0.26	3.8	6	0.18
Wong et al. (2008)	10	1	0.10	10.0	–	–
**Total (all included studies)**	**943**	**Min. 286**	**0.30**	**3.3**	**Min. 90**	**0.14**

Abbreviation: Min., minimally.

a If only patient number or number of surgeries was reported, the other was assumed to be equal.

b In several studies, it was unclear if additional patients had a given complication, beyond those reported (“Min.”).

c Some studies reported only patient counts for reoperation, not if any had multiple (“Min.”).

d 26 patients had complications, but the number per patient was not specified.

#### Sternal pseudoarthrosis (non-union)

Only 1 study^30^ reported on sternal pseudoarthrosis (non-union), with a prevalence of 0.07 event per surgery (**Table 3**). No diagnostic criteria were provided.

**Table 3.** Overview of Sternal Complications

	Number of included studies reporting outcome	**Number of clamshell surgeries conducted by these studies** ^a^	**Total number of sternal complications** ^b^	Number of sternal complications per conducted surgery	Range of sternal complications per conducted surgery	Number of conducted surgeries per sternal complication
**Total number of sternal complications**	18/18	830	Min. 286	0.34	0.02-1.35	2.9
**Sternal pseudoarthrosis/non-union**	1/18	15	1	0.07	–	15.0
**Sternal dehiscence**^c^	9/18	569	Min. 93	0.16	0.00-0.43	6.1
**Sternal separation**^c^	2/18	149	26	0.17	0.14-0.40	5.7
**Sternal override**^c^	6/18	309	Min. 57	0.18	0.00-0.32	5.4
**Sternal wire problems**	4/18	277	24	0.09	0.01-0.21	11.5
**Wire migration**	2/18	101	8	0.08	0.04-0.12	12.6
**Other mechanical wire problems**	2/18	176	16	0.09	0.01-0.21	11.0
**Sternal infection**	12/18	513	Min. 27	0.05	0.00-0.20	19.0
**Superficial sternal wound infection**	2/18	156	Min. 2	0.01	0.01-0.02	78.0
**Deep sternal wound infection**	4/18	213	Min. 11	0.05	0.02-0.07	19.4
**Infected sternal** **haematoma**	2/18	72	2	0.03	0.02-0.05	36.0
**Presternal abscess**	1/18	68	1	0.01	–	68.0
**Sternal osteomyelitis**	1/18	68	1	0.01	–	68.0
**Sternal tissue necrosis**	1/18	68	2	0.03	–	34.0
**Sternal instability**	6/18	262	13	0.05	0.01-0.11	20.2
**Sternal malunion**	3/18	196	3	0.02	0.00-0.05	65.3
**Sternal deformity**	2/18	71	15	0.21	0.10-0.50	4.7
**Chronic sternal pain**	5/18	186	Min. 49	0.26	0.07-0.57	3.8
**Sternal clicking sensation**	2/18	70	6	0.09	0.02-0.25	11.7
**Restricted physical activity**	0/18	–	–	–	–	–
**Sternal reoperation due to complication**^d^	12/18	655	Min. 90	0.14	0.02-0.29	7.3
**Long-term removal of sternal OSM**	0/18	–	–	–	–	–

Abbreviations: Min., minimally; OSM, osteosynthesis materials.

a If only patient number or number of surgeries was reported, the other was assumed to be equal.

b In several studies, it was unclear if additional patients had a given complication, beyond those reported (“Min.”).

c Two studies reported sternal override and separation as subtypes of dehiscence; others treated them as distinct. This table shows them separately.

d Some studies reported only patient counts for reoperation, not if any had multiple (“Min.”).

#### Sternal dehiscence

Nine studies^1^^,^^3^^,^^5^^,^^15^^,^^17^^,^^19^^,^^21^^,^^22^^,^^29^ described sternal dehiscence. Definitions ranged from “sternal dehiscence” to “sternal disruption”^22^ and “sternal override or separation”.^3^^,^^21^^,^^29^ Although the latter 3 studies defined override and/or separation as subtypes of sternal dehiscence, other authors reported dehiscence, override, and separation as separate entities. Therefore, these outcomes are displayed here as separate complications.

Three studies^3^^,^^21^^,^^29^ defined lateral X-ray or chest CT-scan as diagnostic medium, but only 1 study^3^ defined diagnostic criteria. Meyers et al mentioned sternal dehiscence as one of their outcomes, without providing specific results.^7^

There were at least 93 cases of dehiscence among 569 clamshell surgeries, or 0.16 event per surgery. In individual studies, prevalence ranged from 0.00^19^ to 0.43^3^ event per surgery (**Table 3**).

#### Sternal separation and sternal override

Sternal separation and sternal override were reported by 2 studies^3^^,^^25^ and 6 studies^3–6^^,^^25^^,^^29^, respectively. Meyers et al included sternal override in their complications, without reporting separate results.^7^

Most studies did not define diagnostic criteria, except Koster et al (lateral X-ray)^3^ and Fuller et al (physical examination using sternal instability scale).^25^ Definitions by Koster et al and Fuller et al directly contradicted each other, with sternal override defined as either displacement with partly-maintained overlap^3^ or complete separation with overriding parts^25^ and vice versa.

In total, 26 sternal separations were reported among 149 surgeries (0.17 event per surgery), with 0.14^3^ and 0.40^25^ events per surgery in the 2 individual studies. Sternal override occurred at least 57 times in 309 surgeries—0.18 event per surgery, range 0^25^-0.32^4^ (**Table 3**).

#### Sternal wire problems

Sternal wire problems were reported by 4 studies,^5^^,^^7^^,^^19^^,^^21^ and subdivided into wire migration (reported by 2 studies^5^^,^^7^) and other mechanical wire problems (also reported by 2 studies^19^^,^^21^). No diagnostic criteria were provided. Notably, Costa et al reported a wire complication 3 years postoperatively.^19^

In total, 24 cases of wire problems occurred in 277 surgeries (0.09 event per surgery), with wire migration occurring in 8/101 surgeries—0.08 event per surgery; range 0.04^5^-0.12^7^—and other mechanical wire problems in 16/176—0.09 event per surgery; range 0.01^19^-0.21^21^—(**Table 3**).

#### Sternal infection

Twelve^1^^,^^5^^,^^15^^,^^17^^,^^19^^,^^21^^,^^22^^,^^25–27^^,^^29^^,^^30^ studies assessed sternal infection. Two studies^7^^,^^28^ mentioned infection as outcome parameter without reporting details, while in other studies^15^^,^^17^^,^^19^^,^^29^ it was unclear whether certain patients with infection were not explicitly reported (for instance, only deep infections requiring reoperation were reported, or superficial infections were grouped with other minor complications and could not be analysed separately). Diagnostic criteria for (specific types of) infection were not provided.

At least 27 cases of sternal infection occurred among 513 surgeries, equalling 0.05 event per surgery—range 0.00^30^-0.20^25^ in individual studies. Specifically, rate of superficial sternal wound infection was at least 2 out of 156 surgeries—0.01 event per surgery; 0.01^19^ and 0.02^5^ in the 2 reporting studies. At least 11 cases of deep sternal wound-infection occurred among 213 surgeries—0.05 event per surgery; range 0.02^29^-0.07^21^. Infected sternal haematoma was reported in 2 of 72 surgeries—0.03 event per surgery; 0.02^5^ and 0.05^1^ in individual studies. Sternal abscess and sternal osteomyelitis were only reported by 1 study^17^ and both occurred in 1/68 surgeries (0.01 event per surgery) (**Table 3**).

#### Sternal tissue necrosis

Tissue necrosis was reported by 1 study^17^ and occurred in 2/68 surgeries (0.03 event per surgery) (**Table 3**). No definition was provided.

#### Sternal instability

Sternal instability was reported by 6 studies.^5^^,^^7^^,^^15^^,^^17^^,^^22^^,^^26^ Diagnostic criteria were not specified. 13 cases of sternal instability occurred in 262 surgeries, corresponding to 0.05 events per surgery—range 0.01^17^-0.11^26^ (**Table 3**).

#### Sternal malunion

Three studies reported sternal malunion, without defining criteria. Malunion occurred in 3 cases among 196 surgeries—0.02 event per surgery, range 0^19^-0.05^1^ (**Table 3**).

#### Sternal deformity

Sternal deformity was described by 2 studies.^7^^,^^25^ Fuller et al defined it as step-deformity on palpation,^25^ while Meyers et al did not provide criteria.^7^ Deformity occurred 15 times in 71 surgeries—0.21 events per conducted surgery; 0.10^7^ and 0.50^25^ in individual studies (**Table 3**).

#### Chronic sternal pain

Five studies^1^^,^^4^^,^^6^^,^^29^^,^^31^ assessed chronic sternal pain. However, in 2 studies^6^^,^^29^ it was unclear whether additional patients experienced chronic pain, as pain was only reported in patients with other sternal complications. Furthermore, 2 studies^7^^,^^25^ reported sternal pain as an outcome without providing separate results.

Chronic pain was defined by Klinger et al as score >0 on the Numeric Rating-Scale (NRS) >2 months postoperatively^31^ and by Boudreaux et al as pain requiring narcotics >6 months postoperatively.^1^ Other authors did not provide criteria.

At least 49 cases of chronic sternal pain occurred in 186 surgeries, amounting to 0.26 events per surgery—range 0.07^29^-0.57^31^ (**Table 3**).

#### Sternal clicking sensation

Two studies^5^^,^^25^ reported on sternal clicking sensation (unknown diagnostic criteria), with 6 occurrences in 70 surgeries—0.09 event per surgery; 0.02^5^ and 0.25^25^ in individual studies (**Table 3**).

#### Restricted physical activity due to sternal complication

None of the included studies reported on restricted physical activity (**Table 3**).

### Sternal reoperations

#### Reoperation due to sternal complication

Twelve studies^1^^,^^3–7^^,^^15^^,^^17^^,^^19^^,^^21^^,^^29^^,^^30^ reported on sternal reoperations due to sternal complications. Reoperation primarily consisted of sternal wound debridement, extraction of migrated or broken wires, and/or re-approximation using varying osteosynthesis materials.

Several studies^1^^,^^3–5^ reported only the number of patients undergoing sternal reoperation, with unclear total numbers of reoperations; therefore, the number of reoperations reported in this review reflects a minimum. In addition to reoperations, many studies reported additional patients receiving medical treatment and/or local debridement for sternal wound problems.

At least 90 reoperations were performed after 655 surgeries, amounting to 0.14 sternal reoperations per clamshell surgery—range 0.02^19^-0.29^21^—or 7.3 surgeries performed for one sternal reoperation to occur (**Table 3**).

#### Long-term removal of sternal osteosynthesis material

None of the included studies reported on removal of sternal osteosynthesis material on long term (**Table 3**).

### Correlation between patient characteristics and sternal complications

Four studies^1^^,^^5^^,^^17^^,^^21^^,^^29^ reported some characteristics of patients with sternal complications, but 1 study^29^ lacked full-cohort data for comparison. Among 4 patients with sternal complications, Boudreaux et al^1^ reported numerically higher BMI and smoking history, but lower diabetes, preoperative steroid use, and osteoporosis rates than the whole cohort (no *P*-values).

Two studies conducted logistic regression analysis, although numbers were small. Lung allocation score and clamshell incision were significantly associated with major sternal wound complications in a mixed analysis of clamshell surgeries and median sternotomies.^17^ Osteoporosis and conventional wiring closure were significantly associated with sternal dehiscence after clamshell surgery; in subgroup analysis, sternal dehiscence rate was significantly higher in patients with osteoporosis than in patients without (78% vs 25%).^21^

### Correlation between sternal complications and other treatment outcomes

In-hospital mortality was 50% among 6 reoperated patients with sternal dehiscence (26.5% in the whole clamshell cohort)^15^ and 33% among 9 reoperated patients with major sternal complications.^17^ Three studies reported 0% in-hospital mortality: among 2 patients with sternal complications,^6^ among 15 clamshell patients (1 with complications),^30^ and among 59 patients (4 with severe dehiscence; however, 10 patients with unknown complication status were excluded before analysis).^29^

Three studies^1^^,^^6^^,^^29^ reported good long-term outcomes after sternal reoperation; 1 study^26^ reported no long-term problems after early sternal instability.

## DISCUSSION

This systematic literature review found 286 sternal complications after 830 clamshell thoracotomies in adults undergoing bilateral lung or heart-lung transplantation, equating to 0.34 event per clamshell incision. In individual studies, prevalence ranged widely between 0.02 and 1.35 event per surgery. Notably, most included studies were of poor quality, with many sternal complications either ambiguously or not reported, and definitions of complications often sparse or absent. These deficiencies impacted not only the quality of individual studies but also comparability between studies. Due to unclear and incomplete reporting of complications in many articles, complication rates reported in this review are likely an underestimation of the true rates. In several studies, complications were mentioned but not quantified, and various complications were only reported by few studies, raising the possibility of underreporting. Nevertheless, the present review underscores the high number of sternal complications after clamshell thoracotomy.

The significance of these sternal complications should not be underestimated. In this review, 0.14 operative re-interventions were performed per clamshell surgery, and many studies reported medical treatment and/or local wound debridement in additional patients. In-hospital mortality after sternal complications ranged widely between 0% and 30%-50% of patients requiring sternal reoperation. While no firm conclusions could be drawn due to small sample sizes and incomplete reporting by many studies, these high mortality rates underscore the potentially severe morbidity associated with sternal complications.

Besides impacting patients’ postoperative functioning and quality-of-life, complications and their treatment pose considerable financial burdens. For instance, although no financial analyses have been conducted on sternal complications after clamshell thoracotomy, a systematic literature review on data from 14 (mostly Western) countries revealed that sternal wound infections after coronary artery bypass-grafting accounted for a median cost increase of $13 995 per infection, with increased length of hospital stay as largest component.^32^ In the United States, estimated additional costs range from €36 768^32^ to €56 003^33^ per deep sternal wound infection. Chronic postoperative pain after lung transplantation may lead to increased healthcare costs due to the need for long-term analgesic interventions.^34^

Clamshell sternothoracotomy finds it origins in World War-I^2^ and was historically extensively used for emergency thoracotomies and a wide array of elective cardiac surgeries.^2^^,^^12^ Due to significant postoperative complications, its use in elective surgery declined until Pasque et al and Bisson et al pioneered the bilateral sequential lung transplantation through “cross-bow”-incision^35^ or “horizontal sternal bithoracotomy”,^36^ as alternatives for en-bloc double lung transplantation through median thoracotomy.^2^^,^^7^^,^^11^^,^^12^^,^^35–37^ Nowadays clamshell thoracotomy finds its application primarily in bilateral lung transplantations, heart-lung transplantations, and resection of extensive thoracic and/or mediastinal tumors.^2–4^^,^^7–10^^,^^38^

The clamshell incision remains highly relevant because of its superior visualisation of thoracic organs and access to the posterior mediastinum,^2–4^^,^^7–9^^,^^11–13^^,^^16^^,^^22^^,^^39^ and rapid access for cardiopulmonary bypass through central cannulation in case of haemodynamic compromise.^3^^,^^4^^,^^8^^,^^9^^,^^14^^,^^22^

However, the significant postoperative complications remain problematic. Beyond their direct clinical consequences, these complications may lead to prolonged (epidural) analgesia,^11^^,^^40^^,^^41^ reduced pulmonary and chest wall-functioning,^14^^,^^18^^,^^20^^,^^41–44^ prolonged mechanical ventilation time,^15^ more pulmonary infections,^15^^,^^40^ prolonged hospital and ICU-stay,^7^^,^^15^^,^^41^^,^^42^ delayed patient mobilisation,^42^ and slower general recovery.^11^^,^^14^^,^^16^^,^^22^ Moreover, chronic pain could significantly impact patients’ daily functioning and quality-of-life.^34^^,^^45^^,^^46^

Several factors play a role in the high prevalence of sternal complications. Inherently, the clamshell incision, with the extensive surgical trauma including ligation of bilateral mammary arteries and accessory respiratory muscles,^4^^,^^9^^,^^12^^,^^15^^,^^17^^,^^18^^,^^34^ increases the risk of sternal healing complications.^4^^,^^12^^,^^15^^,^^17^ Moreover, altered chest wall mechanics in the postoperative period (increased outward translational chest movement with paradoxical abdominal movement)^3^^,^^15^ result in angular anterior displacement of the distal sternal segment^1^^,^^7^^,^^19^ with increased tension on the sternal fixation site. Traditional wire-cerclage does not prevent this anteroposterior movement^3^^,^^7^^,^^19^ and the overlapping surface between proximal and distal sternal parts after transverse sternotomy is inherently limited.^15^ Additionally, history of smoking, chronic use of immunosuppressive medication, diabetes, osteoporosis, obesity, and malnutrition increase risks of complications in lung transplantation patients.^1^^,^^5–8^^,^^15^^,^^19–21^^,^^40^^,^^43^ In this review, one study^21^ reported a significant association between osteoporosis and sternal complications, while another study^1^ suggested potential influence of smoking or BMI. However, as reports studied different factors and associations were contradicted by other studies,^1^^,^^17^ no firm conclusions could be drawn.

The general complication rate in this review (0.34 event per surgery) is likely an underestimate. Reported complication rates are heavily influenced by differences in patient populations, definitions, sternal closure techniques, and study quality. Notably, important patient characteristics such as age, aetiology, immunosuppressive medication, and previous surgery differed significantly between included studies and were often not reported. Definitions and follow-up length varied considerably. Moreover, several studies mentioned certain complications as one of their outcome measures but failed to report specific results. In general, chronic sternal pain (0.26 events per surgery) and sternal deformity (0.21) occurred frequently, while sternal non-union (0.07), sternal infection (0.05), sternal instability (0.05), and sternal malunion (0.02) were much less frequent. Some specific sternal complications were assessed by few studies, such as sternal non-union, sternal separation, sternal deformity, and specific sternal infections; therefore, their reported prevalence in this review is likely not representative.

To prevent sternal complications, several sternal closure techniques were developed as an alternative to traditional sternal wire-cerclage: reinforced sternal wiring,^5^^,^^21^ crossed sternal wiring,^3^ sternal plating,^15^^,^^22^^,^^43^ plate-and-band,^1^ sternal FiberTape,^5^ absorbable sternal pins,^6^ peristernal cables,^47^ Steinmann pins,^7^^,^^12^ and K-wires^7^^,^^12^ have been proposed. Other authors have developed alternative transverse sternotomy techniques, such as modified transverse sternotomy with quasisternal lock-in mechanism,^19^ bevelled sternotomy,^48^ and sternal notching.^49^

In a biomechanical study, reinforced wiring was significantly stronger than conventional wiring in both anterior-posterior shear and longitudinal distraction.^21^ Clinically, alternative closure and sternotomy-techniques have been associated with decreased sternal complications,^1^^,^^3^^,^^5^^,^^19^^,^^22^ but research is limited. Sternal plating might give better results than reinforced wiring.^5^ However, one study reported that sternal complications did not decrease with sternal plating and preferred a sternal-sparing surgical approach.^15^ Potential downsides of alternative closure techniques could be increased costs^3^ and removal of osteosynthesis materials on the long-term,^3^^,^^47^ but have not been properly researched. Larger cohort studies with appropriate statistical correction for potential confounding factors are sparse.^3^^,^^21^ In one regression analysis, conventional wiring and osteoporosis were significant risk factors for sternal dehiscence.^21^ While no difference in sternal dehiscence was found between conventional and reinforced wiring in the general population, reinforced wiring significantly reduced sternal dehiscence in patients with osteoporosis,^21^ highlighting the importance of patient selection and risk stratification.

Unfortunately, due to wide heterogeneity between studies and widespread subtle differences between the modified closure techniques applied in each study, no subgroup analysis could be conducted for different closure techniques.

Despite promising results, several authors argued that alternative closure techniques will not have sufficient effect to counteract sternal complications and advocated alternative incisions without sternal division.^4^^,^^15^

Alternative surgical approaches are sternal-sparing bilateral anterior thoracotomy^7–9^^,^^15^^,^^18^^,^^28^^,^^42^^,^^50–56^—sometimes video-assisted,^16^^,^^40^ median sternotomy,^4^^,^^9^^,^^11^^,^^17^^,^^39^^,^^50^^,^^57–59^ and minimally invasive or robot-assisted mini-thoracotomy.^14^^,^^41^^,^^60^ In general, these approaches have yielded operative and pulmonary functional results that were equivalent or more favourable than clamshell incisions, and are considered safe and effective alternatives to clamshell thoracotomy.^4^^,^^7^^,^^8^^,^^14^^,^^15^^,^^18^^,^^41^^,^^42^^,^^51^^,^^52^^,^^55^^,^^57^^,^^58^^,^^60^

Sternal complications are absent with sternal-sparing thoracotomy but are also significantly less prevalent with median sternotomy.^4^^,^^9^^,^^17^ Moreover, both median sternotomy and bilateral thoracotomy are associated with fewer (major) wound complications,^7^^,^^8^^,^^17^^,^^18^^,^^57^ decreased postoperative pain,^4^^,^^11^^,^^18^^,^^57^^,^^59^ improved pulmonary and chest wall function,^4^^,^^9^^,^^15^^,^^17^^,^^18^^,^^52^ less blood loss and transfusion requirements,^52^^,^^57^^,^^61^ shorter operation-time,^9^^,^^15^^,^^58^^,^^59^ reduced mechanical ventilation duration,^15^^,^^39^^,^^52^^,^^57^^,^^61^ shorter ICU-stay,^9^^,^^15^^,^^52^^,^^61^ and faster general recovery.^15^^,^^57^ However, both alternative approaches are associated with longer ischaemic time than clamshell incision.^17^^,^^52^^,^^62^ Additionally, other studies found conflicting results, with higher postoperative pain scores after bilateral thoracotomy than clamshell incision^61^ and lower rate of pneumonia^62^ after clamshell. Minimally invasive and robot-assisted mini-thoracotomies are associated with decreased postoperative pain,^14^^,^^41^ improved chest wall function,^14^ shorter ICU and hospital-stay,^14^ and satisfactory postoperative outcomes,^14^^,^^60^ but research to date remains limited.

Others have suggested that within the transplantation population, unfavourable results after clamshell surgery compared with other surgical approaches are confounded by differences in baseline characteristics.^2^ In general, larger (prospective) cohort studies with appropriate correction for confounding factors and randomised trials are lacking.

Choice of surgical approach is multifactorial and depends on patient pathology, anatomy, extracorporeal support requirements, and the surgeon’s experience and preference. Clamshell-incision may still be the preferred approach in case of anticipated difficult dissection,^3^^,^^7^ retransplantation or other major previous thoracic surgery,^3^^,^^17^^,^^40^ severe pleural adhesions (specifically apical and posterior adhesions),^3^^,^^4^^,^^6^^,^^17^^,^^21^^,^^22^^,^^57^^,^^63^ enlarged hilar lymph nodes,^57^^,^^63^ deep hilar structures,^9^^,^^17^ anticipated difficult left hilar access,^7^ anticipated CPB requirement,^3^^,^^6^^,^^7^^,^^21^ narrow chest,^6^^,^^60^ and left-deviated or enlarged heart.^17^^,^^21^ In contrast, patients with minimal adhesions^9^^,^^57^ or high risk for sternal wound complications (such as severe disease or high acuity on the transplant list)^17^ may be preferred candidates for an alternative approach. Robotic lung transplantation through mini-thoracotomy may be feasible when total lung capacity is >3 liters.^60^ Risk assessment based on preoperative CT-scan is considered essential.^17^^,^^63^

While easy access to the heart for cardiopulmonary bypass is one of the main advantages of clamshell sternothoracotomy,^3^^,^^4^^,^^6^^,^^7^^,^^16^ standard CPB use has decreased with use of a double-lumen tracheal tube and single-lung ventilation.^3^^,^^14^^,^^17^^,^^52^^,^^64^ In this review, intraoperative CPB/ECMO use during clamshell thoracotomy varied widely, ranging from 29.6%^26^ to 100%,^4^ reflecting significant differences in practice across centres and surgeons.

CPB has been associated with complications, such as increased risk of primary graft dysfunction^17^^,^^59^^,^^65^ and prolonged mechanical ventilation.^61^^,^^66^ Notably, Marczin et al found that the improved postoperative pulmonary function seen with sternal-sparing bilateral thoracotomy compared to clamshell was negated in patients requiring CPB.^52^ However, long-term risks of CPB remain debated, with conflicting reports.^66^ Moreover, differences in CPB between surgical approaches may be influenced by surgeons’ learning curve of alternative approaches for bilateral lung and heart-lung transplantation.^17^

In median sternotomy, extracorporeal support through CPB or ECMO is typically required.^9^^,^^17^^,^^57^^,^^61^^,^^63^ Two studies reported that CPB time was significantly longer after median sternotomy compared to clamshell surgery.^17^^,^^58^ However, no difference in long-term outcomes were observed,^17^ suggesting that additional CPB use in median sternotomy would counterbalance the decrease in sternal complications after clamshell incision.

Sternal-sparing bilateral thoracotomy provides more limited access to the heart if emergency CPB is needed^8^ and may require conversion to full clamshell incision.^7^^,^^18^^,^^28^^,^^52^ However, other authors have reported safe central cannulation through the aorta or right atrium using bilateral thoracotomies without sternal division,^3^^,^^4^^,^^40^^,^^52^ as well as through transcutaneous extracorporeal cannulation^18^ or the femoral approach.^3^^,^^8^^,^^16^^,^^18^^,^^64^ Some prefer elective CPB installation presurgery, making emergency conversion unnecessary.^7^

Recently, the use of ECMO instead of conventional CPB has increased and may have more favourable postoperative outcomes.^13^^,^^16^^,^^51^^,^^54^^,^^60^^,^^67^ Thus, when considering alternative surgical approaches for bilateral lung or heart-lung transplantation, patients with high likelihood of requiring extracorporeal support (e.g., significant pulmonary hypertension) could be candidates for median sternotomy, while low-risk patients or patients eligible for ECMO might undergo sternal-sparing bilateral thoracotomy or mini-thoracotomy.

The results of this study should be interpreted in light of several limitations. First, no randomised controlled trials have been published on this topic, and only cohort studies were included in this review. Second, most included studies were of poor quality. Definitions of complications were often lacking, and results were frequently succinct and open to multiple interpretations, leading to likely underestimation of complication rates in this review. Third, 2 cases of retransplantation, which may have higher complication rates than first transplantations, could not be excluded from this review due to a lack of separately reported results. Finally, meta-analysis of sternal complications and pooling of sternal closure techniques could not be performed due to significant heterogeneity between studies.

Despite these limitations, this systematic review offers important strengths. It is the first to specifically focus on sternal complications following clamshell thoracotomy in adult bilateral lung and heart-lung transplant recipients. The literature search applied no time restrictions, and all study designs were included, ensuring that all relevant studies on this topic were captured. The reported complication rate provides a quantitative estimate of the true rate and already warrants concern. Given the rising transplant volumes and the substantial morbidity associated with sternal complications, the findings of this review are highly relevant.

The high sternal complication rate, variability across studies, and the impact of comorbidities, closure techniques, and surgical approach highlight the critical importance of accurate patient selection and risk stratification to reduce unnecessary clamshell thoracotomy. Advances in medical treatment and technologies will lead to increasingly vulnerable patients undergoing transplantation in the future, further increasing postoperative complications.^2^^,^^13^

The results of this review could serve as a benchmark for future studies, which should emphasise development, implementation, and evaluation of modified sternal closure techniques to reduce sternal complications after clamshell surgery. Furthermore, future studies should focus on optimising and expanding alternative surgical approaches for bilateral lung or heart-lung transplantation. These studies should prioritise risk stratification to identify candidates for a sternal-sparing approach and refine technical aspects to expand the pool of patients eligible for a sternal-sparing procedure, ultimately reducing the need for clamshell thoracotomy.

In future studies, larger patient cohorts and prospective and/or randomised designs are essential. In this review, only 1 study^19^ included more than 100 patients. Uniform definitions for sternal closure techniques and complications are crucial to enable adequate comparability between studies and patient subgroups. Authors should consistently report on all relevant sternal complications, along with essential preoperative and perioperative variables, such as postoperative drain duration. Long-term sternal outcomes should be prioritised, and patient-reported outcomes may provide additional value in identifying the optimal surgical approach.

## CONCLUSION

In conclusion, sternal complications following the clamshell approach for bilateral lung or heart-lung transplantation represent a significant burden, both in terms of patient morbidity and increased healthcare costs. This systematic literature review can serve as a benchmark for future studies on the safety and efficacy of alternative sternal closing techniques, as well as alternative surgical approaches aimed at reducing sternal complications.

## Supplementary Material

ezaf318_Supplementary_Data

## Data Availability

The data underlying this article (review protocol, extracted data from included studies, data used for all analyses) will be shared on reasonable request to the corresponding author.
