# Supplementary material for: Sternal Complications After Clamshell Surgery for (Heart-)Lung Transplantation—A Systematic Literature Review
Source: Eur J Cardiothorac Surg. 2025 Sep 23;67(11):ezaf318. doi: 10.1093/ejcts/ezaf318 (PMC12582368; doi:10.1093/ejcts/ezaf318)
Supplement: ezaf318_Supplementary_Data [file ezaf318_supplementary_data.zip › Supplementary Tables S1 and S2.docx]

# **Supplementary Table S1. Quality assessment of included studies**

| **Supplementary table S1. Quality assessment of included studies** | | | | | | | | | | | | | | |
| --- | --- | --- | --- | --- | --- | --- | --- | --- | --- | --- | --- | --- | --- | --- |
| **Study** | **Risk of bias criteria** | | | | | | | | | | | | **Quality score** | **Quality rating** |
|  | Research question / objective clearly stated | Study population clearly defined | Participation rate >50% | Recruitment from similar populations; criteria prespecified | Sample size  justified (power, variance, effect estimates) | Exposure  measured before outcomes | Sufficient timeframe to  detect outcomes | Exposure clearly defined, valid, reliable,  consistent | Outcomes clearly defined, valid, reliable,  consistent | Blinding of outcome  assessors | Loss to follow-up after baseline  <20% | Confounding variables  adjusted for |  |  |
| Boudreaux et al. (2021) | Yes | Yes | Yes | Yes | No | Yes | Unclear | Yes | No | Unclear | Unclear | NA | 6 | Poor |
| Brown et al. (1996) | Yes | No | Unclear | Unclear | No | Yes | Yes | Yes | No | Unclear | Unclear | NA | 4 | Poor |
| Coloni et al. (2004) | No | No | Yes | Yes | No | Yes | Unclear | No | No | Unclear | Unclear | NA | 3 | Poor |
| Costa et al. (2015) | Yes | No | Yes | Yes | No | Yes | No | Yes | No | Unclear | Yes | NA | 6 | Poor |
| Coster et al. (2022) | Yes | Yes | Yes | Yes | No | Yes | Unclear | Yes | No | Unclear | Unclear | NA | 6 | Poor |
| Elde et al. (2017) | Yes | Yes | Yes | Yes | No | Yes | Yes | No | Yes | Unclear | Unclear | Yes | 8 | Fair |
| Force et al. (2006) | Yes | Yes | Yes | Yes | No | Yes | Unclear | Yes | No | Unclear | Unclear | NA | 6 | Poor |
| Fuller et al. (2018) | Yes | No | Unclear | Yes | No | Yes | Unclear | Yes | Yes | Unclear | Unclear | NA | 5 | Poor |
| Kaiser et al. (1991) | No | Yes | Yes | Yes | No | Yes | Unclear | Yes | No | Unclear | Unclear | NA | 5 | Poor |
| Klinger et al. (2020) | Yes | Yes | No | Yes | No | Yes | Yes | Unclear | Yes | Unclear | No | No | 6 | Poor |
| Koster et al. (2013) | Yes | Yes | Yes | Yes | No | Yes | Yes | Yes | Yes | Unclear | Yes | Yes | 10 | Good |
| Macchiarini et al. (1999) | Yes | Yes | Yes | Yes | No | Yes | Unclear | Yes | No | Unclear | Unclear | NA | 6 | Poor |
| Meyers et al. (1999) | No | No | Yes | Yes | No | Yes | Unclear | Yes | Unclear | Unclear | Unclear | NA | 4 | Poor |
| Motomura et al. (2011) | No | Yes | Yes | Yes | No | Yes | Yes | Yes | Unclear | Unclear | Unclear | NA | 6 | Poor |
| Olland et al. (2017) | No | Yes | Yes | Yes | No | Yes | Unclear | Unclear | No | Unclear | Unclear | NA | 4 | Poor |
| Oto et al. (2007) | Yes | Yes | Yes | Yes | No | Yes | Yes | Yes | Yes | Unclear | Yes | Yes | 10 | Good |
| Park et al. (2023) | Yes | Yes | Yes | Yes | No | Yes | Unclear | Yes | No | Unclear | Unclear | NA | 6 | Poor |
| Wong et al. (2008) | No | Yes | Yes | Yes | No | Yes | Yes | Yes | No | Unclear | Unclear | NA | 6 | Poor |
| *Quality rating: low (0-6 points), fair (7-9 points), good (10-12 points). NA, not applicable.* | | | | | | | | | | | | | | |
| *Quality assessment based on the National Heart, Lung and Blood Institute of National Institutes of Health (NIH) quality assessment tool for observational cohort studies. Two criteria from the NIH tool (different levels of exposure; exposure assessed more than once over time) were left out since they are not applicable to clamshell surgery. Therefore, each study could obtain a maximum of 12 points.* | | | | | | | | | | | | | | |

# **Supplementary table S2. Preoperative and perioperative patient characteristics**

| **Supplementary table S2. Preoperative and perioperative patient characteristics** | | | |
| --- | --- | --- | --- |
|  | **Number of included studies reporting the variable** | **Number of patients included in these studies^a^** | **Reported range  across studies** |
| **Preoperative characteristics** |  |  |  |
| **Female (%)^b^** | 10/18 | 467 | 32.0-75.0 |
| **Mean age in years (±SD)^c^** | 9/18 | 417 | 40(±11)-54(±9.74) |
| **Mean BMI (±SD)^c^** | 4/18 | 194 | 22.3(±0.5)-24.6(±4.2) |
| **Osteoporosis (%)^b^** | 2/18 | 92 | 22.7-35.7 |
| **Diabetes (%)^b^** | 3/18 | 160 | 2.9-40.9 |
| **Preoperative immunosuppressive medication (%)^b^** | 3/18 | 160 | 34.3-61.8 |
| **Smoking (%)^b^** | 3/18 | 160 | 31.8-51.5 |
| **Malnutrition (%)^b^** | 0/18 | - | - |
| **Previous surgery at same site (%)^b^** | 4/18 | 135 | 0.0-30.0 |
| **Etiology (%)^b.d^** | 11/18 | 499 |  |
| **COPD** | 7/18 | 393 | 8.8-41.9 |
| **Cystic fibrosis** | 7/18 | 368 | 20.0-100.0 |
| **Interstitial lung disease** | 4/18 | 212 | 3.7-70.6 |
| **Pulmonary fibrosis** | 3/18 | 188 | 18.6-42.0 |
| **Pulmonary hypertension** | 3/18 | 117 | 8.6-54.1 |
| **Bronchiolitis obliterans** | 3/18 | 71 | 2.9-10.0 |
| **Emphysema** | 2/18 | 37 | 30.0-66.7 |
| **Bronchiectasis** | 2/18 | 37 | 3.7-20.0 |
| **Parenchymal lung disease** | 1/18 | 37 | 43.2 |
| **Lymphangioleiomyomatosis** | 1/18 | 10 | 10.0 |
| **Histiocytosis X** | 1/18 | 10 | 10.0 |
| **Tuberous sclerosis** | 1/18 | 10 | 10.0 |
| **ARDS** | 1/18 | 34 | 8.8 |
| **Eisenmenger's disease** | 1/18 | 37 | 2.7 |
| **Other (unspecified)** | 5/18 | 194 | 8.8-45.0 |
| **Perioperative characteristics** |  |  |  |
| **Mean operation time in minutes (±SD)^c^** | 3/18 | 185 | 320(±77)-745.18(±101.76) |
| **Use of intraoperative CPB / ECMO (%)^b^** | 8/18 | 379 | 29.6-100.0 |
| **Mean duration of postoperative drain use in days (±SD)^c^** | 0/18 | - | - |
| **Mean length of hospital stay in days (±SD)^c^** | 3/18 | 130 | 24(±8)-74.32(±42.3) |
| **Mean length of ICU stay in days (±SD)^c^** | 4/18 | 259 | 4.1(±3)-21.54(±15.23) |
| *ARDS, acute respiratory distress syndrome. BMI, body mass index. COPD, chronic obstructive pulmonary syndrome. CPB, cardiopulmonary bypass. ECMO, extracorporeal membrane oxygenation. ICU, Intensive Care Unit. SD, standard deviation.* | | | |
| *^a^If only patient number or number of surgeries was reported, the other was assumed to be equal.* | | | |
| *^b^Percentages reflect proportion of included patients.* | | | |
| *^c^Means and SDs are displayed as reported; decimals may vary.* | | | |
| *^d^Values reflect only studies including >1 patient with the given etiology.* | | | |
