# Supplementary material for: Sternal Complications After Clamshell Surgery for (Heart-)Lung Transplantation—A Systematic Literature Review
Source: Eur J Cardiothorac Surg. 2025 Sep 23;67(11):ezaf318. doi: 10.1093/ejcts/ezaf318 (PMC12582368; doi:10.1093/ejcts/ezaf318)
Supplement: ezaf318_Supplementary_Data [file ezaf318_supplementary_data.zip › Supplementary Material.docx]

# **Supplementary Material**

**Appendix A: Pubmed search**

**Appendix B: Embase search**

# **Appendix A: Pubmed search**

(lung transplantation[MeSH Terms] OR "lung transplant*"[Title/Abstract])

AND

(sternotomy[MeSH Terms] OR thoracotomy[MeSH Terms] OR "clamshell"[Title/Abstract] OR “clam shell”[Title/Abstract] OR "sternotom*"[Title/Abstract] OR "thoraco*"[Title/Abstract] OR "sternal division"[Title/Abstract])

*Search conducted on: April 4, 2025*

*Yield: 463 results*

# **Appendix B: Embase search**

(‘lung transplantation’/exp OR ‘lung transplant*’:ab,ti,kw)

AND

(‘sternotomy’/exp OR ‘anterolateral thoracotomy’/exp OR ‘clamshell’:ab,ti,kw OR ‘clam shell’:ab,ti,kw OR ‘sternotom*’:ab,ti,kw OR ‘thoraco*’:ab,ti,kw OR ‘sternal division’:ab,ti,kw)

AND [embase]/lim

NOT 'conference abstract'/it

*Search conducted on: April 4, 2025*

*Yield: 864 results*
